# Association Between Positive Airway Pressure Titration Sleep Data and Therapy Adherence in Patients with Obstructive Sleep Apnea

**DOI:** 10.3390/medicina61091610

**Published:** 2025-09-05

**Authors:** Ji Ho Choi, Sungkyoung Shin, Yeji Lee, Tae Kyoung Ha, Sooyeon Suh

**Affiliations:** 1 Department of Otorhinolaryngology-Head and Neck Surgery, Soonchunhyang University Bucheon Hospital, Soonchunhyang University College of Medicine, 170, Jomaru-ro, Bucheon 14584, Republic of Korea; 2 Department of Psychology, Sungshin Women’s University, 2, Bomun-ro 34da-gil, Seongbuk-gu, Seoul 02844, Republic of Korea; sungyb98@gmail.com (S.S.); yeeyezy@gmail.com (Y.L.); alysuh@sungshin.ac.kr (S.S.); 3 Honeynaps Research and Development Center, Honeynaps Co., Ltd., 529, Nonhyeon-ro, Gangnam-gu, Seoul 06126, Republic of Korea; sean.ha@honeynaps.com

**Keywords:** obstructive sleep apnea, positive airway pressure, adherence, sleep efficiency, wake after sleep onset

## Abstract

*Background and Objectives:* Although numerous studies have explored various predictors of positive airway pressure (PAP) adherence, the potential impact of objective sleep scoring data obtained during PAP titration on adherence has not been thoroughly investigated. The objective of this study was to evaluate the association between objective sleep parameters obtained from PAP titration, including sleep efficiency (SE), wake after sleep onset (WASO), and sleep latency (SL), and short-term PAP adherence in individuals with obstructive sleep apnea (OSA). *Materials and Methods:* A total of 227 individuals with a confirmed diagnosis of OSA underwent overnight PAP titration and were subsequently divided into adherence and non-adherence groups. Baseline demographic characteristics, clinical data, diagnostic polysomnography results, and PAP titration data were obtained for all subjects. Paired sample *t*-tests were utilized to assess differences in sleep parameters between diagnostic polysomnography and PAP titration within each group. Binomial logistic regression was used to evaluate the predictive value of changes in SE, WASO, and SL for PAP adherence and to determine optimal cut-off values. A χ^2^ analysis was conducted to assess the relationship between categorical improvements in SE and WASO and adherence to PAP therapy. *Results:* Among the study cohort, 176 (77.5%) participants were classified as adherent, while 51 (22.5%) participants were classified as non-adherent. SE during PAP titration (83.3 ± 12.6%) was significantly higher compared to baseline polysomnography (80.9 ± 12.4%, *p* = 0.020), and WASO was significantly reduced (63.9 ± 58.9 min vs. 77.7 ± 67.2 min, *p* = 0.016). No significant difference was observed in SL between the two groups. Logistic regression analysis indicated that increased SE (odds ratio [OR]: 1.025, *p* = 0.039) and decreased WASO (OR: 0.994, *p* = 0.027) both served as significant predictors of PAP adherence, but the overall predictive ability of these indicators was modest (area under the curve 0.60 for SE; 0.62 for WASO). The optimal thresholds distinguishing adherence were ΔSE ≥ 2.39% and ΔWASO < −1.5 min. Participants who exhibited improvements in SE (χ^2^ = 5.296, *p* = 0.021) and WASO (χ^2^ = 6.877, *p* = 0.009) demonstrated a significantly higher likelihood of adhering to PAP therapy. *Conclusions:* The findings demonstrate that objective increases in sleep quality, specifically elevated SE and decreased WASO during initial PAP titration, are significantly associated with short-term PAP adherence among patients with OSA.

## 1. Introduction

Obstructive sleep apnea (OSA) is a prevalent sleep-related breathing disorder defined by repeated episodes of either partial or complete upper airway obstruction during sleep [1]. The global burden of OSA is considerable, affecting an estimated 2–4.5% of adults worldwide and showing higher prevalence rates in subgroups such as those with obesity or greater age [2,3]. OSA has been strongly linked to a range of major health complications, including not only cardiovascular diseases such as hypertension, coronary artery disease, and arrhythmias, but also worsening of metabolic conditions like diabetes, a greater likelihood of accidents from pronounced daytime sleepiness, cognitive deficits, and an increased vulnerability to psychiatric conditions, most notably major depressive disorder and posttraumatic stress disorder [4,5,6]. The substantial and wide-ranging impacts of OSA highlight the urgent need for precise and prompt diagnosis, as well as the implementation of appropriate and effective disease management strategies to reduce negative outcomes and significantly improve patients’ overall quality of life [7].

The International Classification of Sleep Disorders, 3rd Edition establishes that OSA diagnosis relies on a combination of clinical evaluation and objective sleep studies [8]. Primary clinical manifestations—including loud snoring, observed apneas, gasping or choking episodes during sleep, and excessive daytime sleepiness—together with symptoms such as reduced concentration, mood changes, and non-restorative sleep, collectively reinforce the clinical suspicion for OSA. A definitive diagnosis necessitates overnight polysomnography or home sleep apnea testing, which objectively records respiratory events such as apneas, hypopneas, and their associated oxygen desaturation episodes. OSA is confirmed when either of the following criteria applies: (1) An apnea-hypopnea index (AHI) ≥ 5 events per hour in patients presenting with typical symptoms or related comorbidities (e.g., hypertension, atrial fibrillation, insulin resistance); or (2) An AHI ≥ 15 events per h, irrespective of clinical symptoms [8].

Multiple therapeutic modalities are employed in OSA management, each presenting unique benefits and drawbacks [9]. Weight reduction achieved through lifestyle modification, pharmacotherapy, or bariatric surgery can markedly alleviate OSA severity by targeting obesity, a major risk factor; however, maintaining weight loss is often difficult and does not guarantee complete OSA resolution in all patients [10]. Positional therapy represents a straightforward and economical method for individuals with positional OSA, though compliance with this intervention tends to diminish over time [11]. Mandibular advancement devices serve as a non-invasive, portable alternative with generally higher adherence rates compared to positive airway pressure (PAP), especially in those with mild to moderate OSA. Nonetheless, these devices may be less effective in lowering the AHI and can be associated with adverse oral or temporomandibular joint effects [12]. Surgical interventions, encompassing upper airway and craniofacial procedures, can enable anatomical correction and long-lasting improvement for selected patients; still, they entail surgical risk and demonstrate inconsistent efficacy among individuals [13,14].

PAP therapy is widely acknowledged as the gold standard treatment for OSA, providing substantial benefits in airway patency, symptom relief, sleep quality, and cardiovascular comorbidities [15,16]. According to the 2009 clinical guideline of the American Academy of Sleep Medicine (AASM), PAP therapy is recommended as the standard treatment for moderate to severe OSA, and may also be considered for mild cases [7]. Notably, the updated AASM clinical practice guideline (2019) recommends PAP therapy for OSA patients presenting with excessive daytime sleepiness, impaired sleep-related quality of life, or comorbid hypertension [17].

The primary objective of PAP titration is to identify the appropriate PAP that will sustain airway patency and prevent sleep-related respiratory events such as apnea and hypopnea [18]. Determining the optimal PAP level during titration is thought to greatly influence both therapy effectiveness and patient adherence with PAP usage [19]. Various parameters assessed during the PAP titration process—including sleep efficiency (SE), wake after sleep onset (WASO), and sleep latency (SL)—may contribute to adherence, though the exact associations remain unclear. Thus, the present study seeks to evaluate how sleep data such as SE, WASO, and SL, derived from PAP titration, affect short-term adherence to PAP therapy among individuals with OSA.

## 2. Materials and Methods

### 2.1. Subjects

This study was conducted at a tertiary university hospital in Korea, where patients diagnosed with OSA consecutively underwent overnight PAP titration between January 2020 and December 2021. All patients were managed by a single, board-certified sleep physician (J.H.C). A retrospective chart review was performed, and the following objective inclusion criteria were used to select the subjects: (1) individuals aged 20 years or older; (2) participants with OSA diagnosed via standard polysomnography, defined as an AHI of 5 or higher with clinically suspected symptoms, or an AHI of 15 or greater independent of symptom presentation; (3) patients who provided consent for PAP therapy for OSA; (4) participants who successfully underwent overnight PAP titration; and (5) those who could be assessed for PAP adherence. PAP adherence was determined according to Korean reimbursement guidelines, which specify a minimum use of ≥4 h per night for at least 70% of nights over any consecutive 30-day period within the first 90 days. Based on these criteria, patients were classified into adherence and non-adherence groups for the purpose of comparative analysis. A total of 227 subjects were finally included in the current study. The study protocol was reviewed and received ethical approval from the Institutional Review Board (IRB) of Soonchunhyang University Bucheon Hospital (IRB No. 2025-04-024). As the study was retrospective and fulfilled the exemption criteria, the requirement for patient consent was waived.

### 2.2. Data Collection

Baseline demographic and clinical variables were obtained at the time of study registration. These included participants’ age, sex, body mass index, neck circumference, and waist-to-hip ratio. Lifestyle characteristics such as current smoking status and alcohol use were also documented. The likelihood of OSA was evaluated utilizing the STOP-Bang questionnaire.

Each participant underwent a thorough physical examination and completed overnight polysomnography in a quiet, dark room with regulated ambient temperature. The polysomnography assessed multiple physiological parameters, including electrooculography, electroencephalography, electrocardiography, chin electromyography, airflow through the nose and mouth (determined by thermistor and nasal pressure sensor), chest and abdominal movement (measured using inductance plethysmography), oxygen saturation (assessed by pulse oximetry), bilateral anterior tibialis electromyography, snoring, and body position. The subjects’ behaviors and sleep postures were continuously monitored and confirmed by a sleep technician utilizing an infrared camera, and all measurements were captured with a digitized polysomnography system (Embla N7000; Natus Medical Inc., San Carlos, CA, USA).

SE was defined as the percentage of total sleep time (TST) relative to total recording time (TRT), calculated as TST ÷ TRT × 100. SL was defined as the time in minutes from lights off to the first recorded sleep stage, representing the duration required to fall asleep. WASO was defined as the total minutes of wakefulness occurring between sleep onset and final awakening, calculated as TRT − SL − TST.

All participants underwent overnight PAP titration with continuous monitoring via polysomnography. Throughout the night, manual titration was performed using the same montage to determine the optimal PAP setting. The standardized PAP titration protocol used in this study included several key elements as follows [18]. During titration, the pressure was gradually increased in increments of at least 1 cm H_2_O, with each step maintained for a minimum of 5 min. Adjustments were guided by the occurrence of respiratory events, defined as two or more obstructive apneas, three or more hypopneas, five or more respiratory effort–related arousals, or at least 3 min of persistent loud or clearly recognizable snoring. This process was continued until a period of at least 30 min free of such events was achieved. The optimal pressure was defined as the minimum pressure that effectively eliminated sleep-related respiratory events and was consistent with the final pressure determined during titration. Residual respiratory events were systematically evaluated by calculating the residual event index using the entire dataset from the PAP titration procedure.

All sleep studies were performed and manually scored by two experienced sleep technologists (RPSGTs) in accordance with the AASM scoring manual and guidelines [18,20]. A single, board-certified sleep physician subsequently reviewed and confirmed all findings to ensure consistency.

### 2.3. Statistical Analysis

Continuous variables are presented as mean ± standard deviation, and categorical variables as percentages. Independent *t*-tests were used to compare continuous variables, while χ^2^ tests assessed categorical variables between the PAP adherence and non-adherence groups. The means of sleep parameters such as SE, WASO, and SL from both polysomnography and PAP titration studies were compared using paired sample *t*-tests. Binomial logistic regression was applied to determine whether changes in each sleep parameter could predict PAP adherence and to identify optimal cutoff points. Differences in sleep parameters were obtained by subtracting polysomnography values from those derived during PAP titration. Based on identified thresholds, participants were grouped, and χ^2^ tests were conducted to compare the distributions of PAP adherence between these groups. All statistical analyses were conducted in R 4.4.1 (R Foundation for Statistical Computing, Vienna, Austria). A *p* value below 0.05 was deemed statistically significant in this study.

## 3. Results

A summary of demographic, clinical, and polysomnographic features comparing PAP adherence and non-adherence groups is provided in Table 1. Participants were divided into adherence (*n* = 176, 77.5%) and non-adherence (*n* = 51, 22.5%) groups according to their PAP usage. Demographic and polysomnographic characteristics were similar between groups except for smoking status, which differed significantly, with a greater proportion of smokers in the non-adherence group. Both groups had comparable demographic profiles, sleep scoring, and rates of respiratory events. The adherence group had a mean age of 49.4 ± 12.3 years and included 151 males (85.8%) and 25 females (14.2%). The non-adherence group had a mean age of 47.4 ± 14.2 years, consisting of 41 males (80.4%) and 10 females (19.6%).

### 3.1. Comparison of Sleep Data Between Overnight Polysomnography and PAP Titration

**Figure 1:**
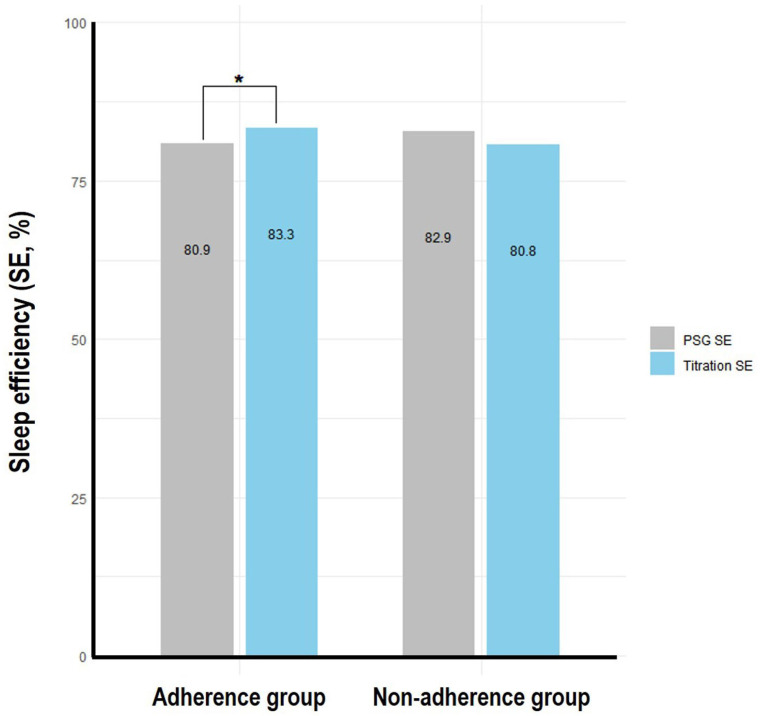
Sleep efficiency (SE) comparison between polysomnography (PSG) and positive airway pressure (PAP) titration in PAP adherence and non-adherence groups (*n* = 227). * Statistically significant difference (*p* < 0.05).

**Figure 2:**
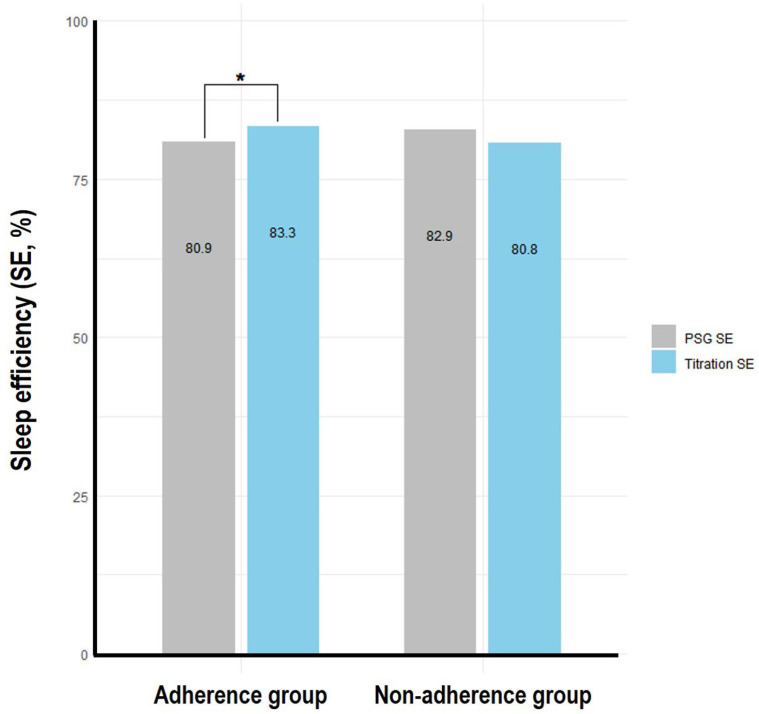
Wake after sleep onset (WASO) comparison between polysomnography (PSG) and positive airway pressure (PAP) titration in PAP adherence and non-adherence groups (*n* = 227). * Statistically significant difference (*p* < 0.05).

Paired sample *t*-tests were utilized to evaluate significant differences in sleep parameters between polysomnography and PAP titration within each group. The findings demonstrated that only the adherence group exhibited significant differences (SE: *t* = −2.340, *p* = 0.020, WASO: *t* = 2.440, *p* = 0.016). Within the adherence group, SE during PAP titration (83.3 ± 12.6%) was notably higher than during polysomnography (80.9 ± 12.4%) (Figure 1), whereas WASO during PAP titration (63.9 ± 58.9 min) was significantly lower compared to polysomnography (77.7 ± 67.2 min) (Figure 2). Nevertheless, there was no significant difference in sleep onset latency between polysomnography and PAP titration in either group.

### 3.2. Impact of Changes in Primary Sleep Parameters on Predicting PAP Adherence and Determination of Optimal Thresholds

Separate binomial logistic regression analyses were performed to assess whether changes in SE, WASO, and SL could predict PAP adherence. Increased SE was significantly linked to higher odds of PAP adherence (odds ratio [OR]: 1.025, 95% confidence interval [CI]: 1.002–1.051, *p* = 0.039). Conversely, greater WASO was significantly linked to reduced odds of adherence (OR: 0.994, 95% CI: 0.989–0.999, *p* = 0.027). SL was not significantly related to adherence (OR: 0.996, *p* = 0.618). These findings established SE and WASO as significant predictors (Table 2). Despite identifying model-based optimal thresholds, the area under the receiver operating characteristic curve (AUC) values remained relatively low, demonstrating modest predictive ability: a difference in SE of 2.39% (AUC = 0.60) and a difference in WASO of −1.5 min (AUC = 0.62).

A χ^2^ test was performed to assess the relationship between SE improvement and PAP adherence (Table 3). In the adherence group, 86 participants (48.9%) met the criteria for improved SE (ΔSE ≥ 2.39%), while 90 participants (51.1%) did not. Among the non-adherence group, 15 participants (29.4%) showed SE improvement, and 36 participants (70.6%) did not. The χ^2^ analysis indicated that participants with improved SE (ΔSE ≥ 2.39%) had a significantly higher likelihood of adhering to PAP therapy (χ^2^ = 5.296, *p* = 0.021), with nearly half (48.9%) in the adherence group showing improvement, compared with less than one-third (29.4%) in the non-adherence group.

Similarly, a χ^2^ test was conducted to evaluate the relationship between changes in WASO and PAP adherence (Table 4). Among those in the adherence group, 101 participants (57.4%) experienced an improvement in WASO (ΔWASO < −1.5), while 75 participants (42.6%) did not show improvement. In contrast, only 18 participants (35.3%) in the non-adherence group had improved WASO, whereas 33 participants (64.7%) did not demonstrate improvement. The χ^2^ test indicated a significant association between WASO improvement (ΔWASO < −1.5) and PAP adherence (χ^2^ = 6.877, *p* = 0.009), revealing that a substantially greater proportion of the adherence group (57.4%) improved compared to the non-adherence group (35.3%).

## 4. Discussion

This study examined the impact of sleep parameters, specifically SE, WASO, and SL, as measured during PAP titration, on short-term adherence to PAP therapy among patients with OSA. Participants were divided into adherence (77.5%) and non-adherence (22.5%) groups based on their PAP usage patterns. With the exception of smoking status, there were no significant differences in demographic or polysomnographic variables between the PAP adherence and non-adherence groups. In the adherence group, SE during PAP titration (83.3 ± 12.6%) was significantly higher than during baseline polysomnography (80.9 ± 12.4%, *p* = 0.020). Moreover, WASO was notably lower during PAP titration (63.9 ± 58.9 min) compared to polysomnography (77.7 ± 67.2 min, *p* = 0.016) for individuals adherent to therapy. Importantly, these significant variations in sleep parameters were found only in the adherence group, whereas sleep onset latency remained unchanged in both groups. The documented changes in sleep scoring data, particularly regarding SE and WASO, during PAP titration compared to diagnostic polysomnography, support the study’s hypothesis that these factors positively affect PAP therapy adherence. Multiple explanations may account for the absence of a statistically significant change in SL observed in this study. First, this outcome likely reflects the primary mechanism of PAP action. PAP specifically targets the reduction in respiratory disturbances (apneas and hypopneas) and limits sleep fragmentation, thereby improving oxygen saturation [18]. These mechanisms predominantly influence sleep maintenance aspects such as SE and WASO, rather than the initiation of sleep. Second, patient adaptation and psychological responses may also contribute. The initial use of a PAP mask and exposure to airway pressure frequently cause discomfort, and some patients experience claustrophobia or anxiety, complicating the process of falling asleep when starting therapy [15]. These issues may offset any favorable effects of PAP on sleep initiation, resulting in considerable inter-individual variability. Alternatively, if SL remained similar to baseline polysomnography, it might indicate that patients successfully adapted to therapy without major initial sleep onset challenges. Third, the nature of baseline SL during diagnostic polysomnography could be a limiting factor. If participants’ baseline SL was already within a typical range (e.g., usually under 30 min), there would be minimal opportunity for further substantial reduction with PAP therapy [8]. Thus, the prospect for notable improvement in SL may have been inherently limited.

In the study, binomial logistic regression analysis demonstrated that higher SE significantly increased the likelihood of PAP adherence (OR: 1.025, *p* = 0.039). In contrast, greater durations of WASO were significantly linked to reduced adherence odds (OR: 0.994, *p* = 0.027). Although SE and WASO emerged as significant predictors, their ability to discriminate between adherent and non-adherent patients was modest, as indicated by relatively low AUC values (0.60 for SE; 0.62 for WASO). The results of the binomial logistic regression analysis offer valuable insights into factors influencing early PAP adherence, emphasizing the importance of objective sleep quality indicators measured during titration. The association between higher SE and shorter WASO with greater odds of PAP adherence highlights the significance of an initial favorable sleep experience with the device. This finding is consistent with the existing literature, which suggests that adherence patterns are established quickly during treatment initiation, often within the first few days or weeks, and that these initial experiences strongly influence long-term compliance [7,15,21]. When patients perceive immediate improvements in sleep quality, evidenced by more consolidated sleep (higher SE) and fewer nocturnal awakenings (lower WASO), they are more likely to recognize tangible benefits from the intervention. This subjective sense of improvement has consistently proven to be a stronger determinant of continued PAP use than objective disease severity alone [22]. Moreover, an initial positive experience may enhance patients’ self-efficacy and expectations for treatment outcomes, thereby strengthening their belief in the device’s utility and their confidence in adhering to therapy [23]. However, it is essential to recognize that the relatively low area under the curve (AUC) values (0.60 for SE; 0.62 for WASO) indicate that, despite statistical significance, these sleep measures on their own have only a modest capacity to predict which individuals will remain adherent. This is expected, as PAP adherence represents a complex behavioral phenomenon influenced by numerous interrelated factors, including demographic variables, device features (e.g., mask comfort, side effects), and psychological or social determinants [15,24]. Consequently, no single variable has consistently been shown to predict adherence by itself. Therefore, while improvements in SE and WASO observed during titration are informative, they should be interpreted as components of a multifaceted evaluation rather than as standalone indicators for adherence prediction.

In this study, the χ^2^ test further indicated that participants who achieved improved SE (ΔSE ≥ 2.39%) were significantly more likely to adhere to PAP therapy (χ^2^ = 5.296, *p* = 0.021). Similarly, significant associations were identified between reductions in WASO (ΔWASO < –1.5 min) and PAP adherence (χ^2^ = 6.877, *p* = 0.009). Logistic regression analyses were performed to evaluate the predictive ability of changes in SE and WASO for PAP adherence; these analyses identified threshold values of a 2.39% improvement in SE and a 1.5 min reduction in WASO that best distinguished adherent from non-adherent participants. The categorical results further support the logistic regression findings by highlighting a pronounced divergence between groups: a substantial proportion of the adherent participants (48.9% for SE, 57.4% for WASO) exhibited these favorable changes during titration, compared to a notably lower percentage in the non- adherent cohort (29.4% for SE, 35.3% for WASO). This observed disparity indicates that a lack of immediate, observable improvements in sleep quality during the titration phase may constitute a significant barrier to later PAP adherence for many patients. Specifically, if patients do not experience prompt benefits, their motivation to persist with PAP therapy, despite challenges such as mask discomfort or the intrusive nature of the device, may decline [15,24]. From a clinical standpoint, these data highlight the importance of optimizing sleep quality during PAP titration and the initial phase of therapy [25]. It is crucial for clinicians to maximize sleep quality in the laboratory setting by ensuring precise mask fitting, individualized pressure adjustments, and addressing any acute sources of discomfort or sleep disturbance [25]. Targeted interventions designed to improve SE and decrease WASO at this stage have the potential to strengthen adherence trajectories. This could include behavioral interventions, such as desensitization for claustrophobia or anxiety—factors well recognized as barriers to effective PAP use [15,24]. Furthermore, the results underscore the importance of early and comprehensive follow-up, preferably within the initial days to weeks following therapy initiation, to address challenges that may threaten a patient’s early engagement and adherence [7,21].

Based on the findings of the study, we propose a clinical practice recommendation for optimizing PAP therapy for patients with OSA. We recommend that in-laboratory PAP titration be regarded not merely as a procedure for determining optimal pressure, but as a crucial strategic process for improving treatment adherence, and that this perspective be incorporated into clinical protocols for PAP therapy initiation. The rationale is as follows: (1) PAP titration can play a significant role in predicting and enhancing future treatment adherence, extending beyond its traditional function of determining optimal pressure. (2) PAP titration serves as a vital opportunity to immediately identify and resolve patient issues, such as mask-fitting problems and psychological discomforts (e.g., claustrophobia or anxiety). (3) During PAP titration, actively improving a patient’s sleep quality by increasing SE and reducing WASO can contribute to better PAP adherence. (4) Specifically, our results indicate that patients who show an improvement in SE of ≥2.39% or a decrease in WASO of ≥1.5 min compared to diagnostic polysomnography are more likely to adhere to PAP therapy. In summary, optimizing mask fitting and identifying a comfortable pressure during PAP titration can facilitate a positive initial experience. This favorable first-phase experience is likely to be a critical factor in enhancing PAP adherence.

This study, although offering important insights into the association between sleep parameters during PAP titration and short-term adherence, has several limitations that must be acknowledged. Firstly, the retrospective single-center design inherently limits the external validity of the results. Moreover, the limited scope of the study warrants cautious interpretation. Secondly, the substantial male predominance in our cohort (over 80% in both groups) may reduce the generalizability of our conclusions to broader patient populations. Thirdly, our exclusive focus on short-term PAP adherence leaves unanswered questions regarding long-term adherence, which continues to pose significant management difficulties in OSA. Fourthly, the potential for behavioral factors such as smoking and alcohol use to act as confounding variables influencing PAP adherence outcomes cannot be excluded. Fifthly, while our study primarily focused on sleep-related predictors of PAP adherence, recent evidence highlights that therapeutic strategies targeting pharyngeal collapse, such as those showed by Lorusso et al., can significantly influence treatment outcomes [26]. Thus, incorporating both polysomnographic predictors and anatomical–functional considerations into future research may provide a more comprehensive framework for understanding variability in treatment response and adherence in OSA therapy. Finally, although SE and WASO emerged as significant predictors, the observed AUC values (0.60 and 0.62, respectively) suggest that these sleep parameters alone offer only limited accuracy in predicting adherence at the individual level. Further clinical research should be conducted to address these limitations and produce findings of greater clinical impact. Despite these limitations, several directions for future research remain evident. Specifically, subsequent investigations comparing the effectiveness of PAP therapy in patients with syndromic versus non-syndromic sleep apnea would be particularly valuable. Current literature lacks direct analyses of treatment outcomes between these two distinct groups [27,28]. Addressing this gap could clarify whether the anatomical characteristics associated with syndromic cases warrant modified or more intensive therapeutic approaches.

## 5. Conclusions

This study demonstrates that objective enhancements in sleep parameters during early PAP titration are significantly related to short-term PAP adherence among patients with OSA. Notably, increased SE and decreased WASO during titration, compared to diagnostic baseline polysomnography, served as principal predictors of adherence. The results highlight the importance of the titration procedure not only for determining optimal pressure, but also as a strategic opportunity to shape a favorable initial experience that may significantly influence ongoing therapeutic engagement.

In clinical settings, these results support the need for enhanced efforts to optimize patients’ sleep quality during in-laboratory PAP titration. This involves careful attention to mask fitting, individualized pressure settings, and proactive strategies to address immediate discomforts or psychological challenges such as claustrophobia or anxiety. Delivering targeted behavioral and supportive interventions informed by real-time sleep data during this key initial period may considerably improve first-phase acceptance and, by extension, support sustained long-term adherence.

## Figures and Tables

**Table 1:** Demographic, clinical, and polysomnographic characteristics compared between positive airway pressure adherence and non-adherence groups (*n* = 227).

	Adherence Group (*n* = 176)	Non-Adherence Group (*n* = 51)	*p*
Demographic characteristics			
Age (years)	49.4 ± 12.3	47.4 ± 14.2	0.352
Sex, male	151 (85.8)	41 (80.4)	0.471
Body mass index (kg/m^2^)	28.9 ± 4.8	30 ± 5.5	0.197
Neck circumference (cm)	39.3 ± 4.5	39.6 ± 3.5	0.560
Waist-to-hip ratio	1.0 ± 0.1	1.0 ± 0.1	0.320
Smoking status (yes)	43 (24.4)	25 (49.0)	0.001 *
Alcohol consumption (yes)	108 (61.4)	34 (66.7)	0.600
Epworth Sleepiness Scale score	9.8 ± 5.2	9.1 ± 4.9	0.409
STOP-Bang questionnaire score	4.7 ± 1.2	4.5 ± 1.2	0.229
Pittsburgh Sleep Quality Index score	5.7 ± 2.4	5.4 ± 2.1	0.419
Polysomnographic parameters			
Total recording duration (min)	425.9 ± 38.5	427 ± 40.8	0.870
TST (min)	341.8 ± 44.4	351.1 ± 41.9	0.173
Sleep efficiency (%)	80.9 ± 12.4	82.9 ± 11.8	0.299
Wake after sleep onset duration (min)	77.7 ± 67.2	62.7 ± 53.6	0.101
Sleep latency duration (min)	10.6 ± 16.9	13.0 ± 15.8	0.356
Stage N1 (% of TST)	29.6 ± 16.8	25.2 ± 15.4	0.081
Stage N2 (% of TST)	41.2 ± 15.2	45.5 ± 15.5	0.084
Stage N3 (% of TST)	3.5 ± 5.9	4.4 ± 6.0	0.355
Stage R (% of TST)	14.8 ± 5.8	15.6 ± 5.5	0.402
Apnea-hypopnea index (events/h)	43.9 ± 25.0	39.1 ± 25.7	0.234
Lowest oxygen saturation (%)	77.6 ± 9.0	77.0 ± 9.6	0.665

The data is presented as mean ± standard deviation or *n* (%). STOP-Bang, snoring, tiredness, observed apnea, high blood pressure, body mass index, age, neck circumference, and male gender; TST, total sleep time. * *p* < 0.05.

**Table 2:** Binary logistic regression analysis of sleep parameters related to positive airway pressure adherence (*n* = 227).

Variable	B ^‡^	Standard Error	OR	95% CI	*p*
SE (%)					
Intercept	1.235	0.161	3.439 †	2.530–4.764	<0.001
SE difference	0.025	0.012	1.025 *	1.002–1.051	0.039
WASO (min)					
Intercept	1.233	0.162	3.432 †	2.522–4.758	<0.001
WASO difference	−0.006	0.003	0.994 *	0.989–0.999	0.027
SL (min)					
Intercept	1.240	0.159	3.456 †	2.550–4.767	<0.001
SL difference	−0.004	0.008	0.996	0.979–1.013	0.618

SE, sleep efficiency; WASO, wake after sleep onset; SL, sleep latency; OR, odds ratio; CI, confidence interval. * *p* < 0.05; ^†^ *p* < 0.001; ^‡^ regression coefficient.

**Table 3:** Chi-square test outcomes for differences in sleep efficiency between positive airway pressure adherence and non-adherence groups (*n* = 227).

	Adherence	Non-Adherence	Total	χ^2^	*p*
Improvement in SE (SE difference * ≥ 2.39)	86 (48.9)	15 (29.4)	101 (44.5)	5.296	0.021
Non-improvement in SE (SE difference * < 2.39)	90 (51.1)	36 (70.6)	126 (55.5)		
Total	176 (77.5)	51 (22.5)	227 (100.0)		

SE, sleep efficiency. * SE difference = titration SE − polysomnography SE.

**Table 4:** Chi-square test outcomes for comparing wakefulness after sleep onset between positive airway pressure adherence and non-adherence groups (*n* = 227).

	Adherence	Non-Adherence	Total	χ^2^	*p*
Improvement in WASO (WASO difference * < −1.5)	101 (57.4)	18 (35.3)	119 (52.4)	6.877	0.009
No improvement in WASO (WASO difference * ≥ −1.5)	75 (42.6)	33 (64.7)	108 (47.6)		
Total	176 (77.5)	51 (22.5)	227 (100.0)		

WASO, wake after sleep onset. * WASO difference = titration WASO − polysomnography WASO.

## Data Availability

The datasets used and/or analyzed during the current study may be provided from the corresponding author, upon appropriate request.

## Abbreviations

The following abbreviations are used in this manuscript:

OSA	Obstructive sleep apnea
AHI	Apnea-hypopnea index
PAP	Positive airway pressure
AASM	American Academy of Sleep Medicine
SE	Sleep efficiency
WASO	Wake after sleep onset
SL	Sleep latency
TST	Total sleep time
TRT	Total recording time
